# Differences in coagulation-relevant parameters: Comparing cryoprecipitate and a human fibrinogen concentrate

**DOI:** 10.1371/journal.pone.0290571

**Published:** 2023-08-30

**Authors:** Sophia Stanford, Ashok Roy, Tom Cecil, Oliver Hegener, Petra Schulz, Anna Turaj, Sean Lim, Emily Arbuthnot

**Affiliations:** 1 Peritoneal Malignancy Institute, Basingstoke and North Hampshire Hospital, Basingstoke, United Kingdom; 2 Octapharma AG, Lachen, Switzerland; 3 Octapharma Pharmazeutika Produktionsges.m.b.H., Vienna, Austria; 4 Faculty of Medicine, Centre for Cancer Immunology, University of Southampton, University Hospital Southampton, Southampton, United Kingdom; University of Ferrara: Universita degli Studi di Ferrara, ITALY

## Abstract

**Background:**

Variable fibrinogen content within cryoprecipitate makes accurate dosing challenging in patients with coagulopathic bleeding, in addition to pathogen transmission risks associated with its administration. Purified and standardized human fibrinogen concentrates (HFCs) represent reliable alternatives. Full cryoprecipitate characterization is required to inform selection of an appropriate fibrinogen source for supplementation therapy.

**Methods:**

Extended biochemical comparison of pooled cryoprecipitate and HFC (*Fibryga*, Octapharma) was performed using commercially available assays to determine levels of variability in cryoprecipitate and HFC. In addition to standard procoagulant factors, measurements included activities of platelet-derived microparticles (PMPs) and plasminogen, and levels of fibrin degradation products.

**Results:**

Cryoprecipitate contains lower fibrinogen levels than HFC (4.83 *vs*.19.73 g/L; p<0.001), translating to approximately half the amount of fibrinogen per standard cryoprecipitate dose (two pools, pre-pooled from five donations each) *vs*. HFC (2.14 *vs*. 3.95 g; p<0.001). Factor XIII (FXIII) levels were also lower in cryoprecipitate *vs*. HFC (192.17 *vs*. 328.33 IU/dL; p = 0.002). Levels of procoagulants in cryoprecipitate, such as von Willebrand Factor (VWF) and factor VIII (FVIII), were highly variable, as was PMP activity. A standard cryoprecipitate dose contains significantly higher levels of measured plasminogen and D-dimer fragments than a standard HFC dose.

**Conclusion:**

The tested HFC is a more reliable fibrinogen and FXIII source for accurate dosing compared with cryoprecipitate. Cryoprecipitate appears considerably less predictable for bleeding management due to wide variation in pro- and anticoagulation factors, the presence of PMPs, and the potential to elevate VWF and FVIII to prothrombotic levels.

## Introduction

Cryoprecipitate is a traditional source of fibrinogen for replacement therapy in the treatment of trauma bleeding cases, post-partum hemorrhage, and in a range of surgical settings [1]. Despite its capacity to replenish plasma fibrinogen levels in patients with coagulopathic bleeding [1], the variable fibrinogen content of cryoprecipitate makes accurate dosing challenging [2]. There are also safety concerns associated with cryoprecipitate, mainly that of pathogen transmission due to the lack of pasteurization/viral inactivation during preparation. This resulted in cryoprecipitate being withdrawn from many Western European countries [1,3,4].

Cryoprecipitate is prepared from fresh frozen plasma donations and, in addition to fibrinogen, contains many other hemostatic coagulation factors, including factor VIII (FVIII), factor XIII (FXIII), von Willebrand factor (VWF), vitronectin, and fibronectin. The exact content of these factors is not standardized and depends upon the method of preparation [3]. A typical bag of pooled cryoprecipitate from National Health Service Blood and Transplant (NHSBT) is prepared from five single donations and contains >700 mg of fibrinogen (mean of 1596 mg) and >350 IU of FVIII (mean of 499 IU) in a typical volume of 100–300 mL (mean of 236 mL) [5]. However, due to natural variation in coagulation factor levels between donors, there is wide variation in fibrinogen and FVIII content between cryoprecipitate units [6]. Other cryoprecipitate constituents are not usually monitored by NHSBT but are also likely to be highly variable. As such, the content of cryoprecipitate is not fully characterized.

In addition to procoagulants, cryoprecipitate is thought to contain other proteins/factors that have the potential to influence hemostasis, including fibrin degradation products (FDPs), plasminogen, and alpha-2 antiplasmin. It is, therefore, unclear whether the complex and variable composition of cryoprecipitate is beneficial for procoagulant activity or contributes to inaccurate dosing and prothrombotic risks to hemostatic therapy.

Purified and standardized human fibrinogen concentrates (HFCs) represent reliable alternatives to cryoprecipitate [7–9]. HFC (*Fibryga*, Octapharma AG) has demonstrated a superior pharmacokinetic profile, as well as comparable safety, efficacy, and clot strength compared with other HFCs [10,11]. In comparison studies, this HFC also demonstrated hemostatic efficacy and noninferiority compared with cryoprecipitate [12,13]. In the FIBRES randomized controlled trial (RCT) of 735 adults who underwent cardiac surgery and developed clinically significant bleeding and hypofibrinogenemia following cardiopulmonary bypass (CPB), HFC (n = 372) was noninferior to cryoprecipitate (n = 363) regarding the number of allogeneic blood components transfused 24-hour post-CPB [12]. In the FORMA-05 RCT, 100% of patients in the per protocol population receiving HFC (n = 21) and cryoprecipitate (n = 22) for treatment of excessive bleeding whilst undergoing cytoreductive surgery (CRS) for pseudomyxoma peritonei achieved hemostatic success [13]. Furthermore, HFC arrived in the operating room 46 minutes faster than cryoprecipitate [13]. No thromboembolic events (TEs) were observed in the HFC group, while seven patients in the cryoprecipitate group experienced seven TEs (pulmonary embolism [n = 5]; deep vein thrombosis [n = 2]) [13]. To better understand potential differences between cryoprecipitate and HFC as used in clinical practice, a detailed analysis of cryoprecipitate composition is required.

Here we provide an extended biochemical analysis of NHSBT cryoprecipitate and HFC (*Fibryga*). We confirm previously documented coagulation factor content and evaluate components not sufficiently characterized in cryoprecipitate previously, including procoagulant activity (rather than concentration) of platelet-derived microparticles (PMPs), levels of FDPs, fibrinopeptide A (FPA), alpha-2 antiplasmin, thrombin anti-thrombin complex (TAT), prothrombin fragment 1+2, and plasminogen activity. Such characterization is required to identify relevant considerations in selecting an appropriate fibrinogen source for supplementation therapy.

## Materials and methods

NHSBT cryoprecipitate was analyzed at the Peritoneal Malignancy Institute at Basingstoke and North Hampshire Hospital, Hampshire Hospital NHS Foundation Trust, UK. HFC (*Fibryga*, Octapharma AG) was analyzed at Octapharma Plasma Research and Development, Vienna, Austria. The primary objective was to characterize NHSBT cryoprecipitate content and compare the variability of the coagulation profile with that of HFC.

### Storage and handling

Pooled cryoprecipitate was purchased from NHSBT (n = 6 bags; all AB+). Each bag contained five individual donor units, which were received frozen and stored at −80°C. For coagulation testing, cryoprecipitate was thawed at 37°C and transferred into 1-mL cryovials before refreezing at −80°C until required for analysis, to avoid freeze-thaw deterioration. Cryoprecipitate bags were each assayed individually as separate samples.

Six HFC vials were reconstituted in the provided 50 mL sterile water according to manufacturer instructions immediately prior to fibrinogen activity analysis. Aliquots were stored at −70°C for further analysis, avoiding repeated freezing/thawing cycles. HFC vials were from six different manufacturing batches (provided as 1 g per vial at 20 mg/mL after reconstitution) and individually assayed.

### Assay methods

Unless otherwise described, all assays were performed according to manufacturer’s instructions. The Sysmex CS series coagulometer (Siemens Healthcare; Erlangen, Germany), and the ACL Elite Pro and DS2 systems (Werfen; Barcelona, Spain) were among those used to perform assays, unless otherwise stated.

### Procoagulant activation markers/components/activity

Clauss fibrinogen activity was measured as previously described [14,15]. Commercially available assays were used to measure fibrinogen, FVIII, and VWF antigen levels, as well as levels of FVIII (FVIII chromogenic and FVIII one-stage [OS]), FXIII, fibronectin, alpha-2 antiplasmin, TAT, prothrombin fragment 1+2, FPA, and PMP activity, as described in the supporting information (S1 File). Both chromogenic and OS assays for FVIII were performed as there has been previously reported discrepancy between results from these assays [16]. For the molar ratio of FPA to fibrinogen, molarity was calculated using the measured FPA and fibrinogen concentrations and the following molecular weights; 1536.6 g/mol for FPA and 340,000 g/mol for fibrinogen.

### Plasminogen and D-dimer fragments

For plasminogen activity, the chromogenic Berichrom Plasminogen (Siemens Healthcare; Erlangen, Germany) and HemosIL Plasminogen (Werfen; Barcelona, Spain) assays were used for cryoprecipitate and HFC, respectively. D-dimer levels were determined using the Asserachrom D-dimer Kit (Diagnostica Stago; Asnières-sur-Seine, France).

### Coagulation factor content per standard dose of cryoprecipitate and HFC

As there is a well-established difference between standard dosing for cryoprecipitate and HFC (10 U *vs*. 4 g, respectively), concentrations of coagulation factors within the two therapy preparations may not reflect coagulation factor levels per standard dose. Therefore, coagulation factor levels for each of the 15 permutations of two cryoprecipitate bags (10 U; e.g., bags 1+2, 1+3, 1+4, etc.) and four HFC vials (4 g; e.g., vials 1+2+3+4, 1+2+3+5, 1+2+3+6, etc.) were determined to provide an assessment of mean coagulation factor levels within a standard dose.

### Statistical analysis

Outcomes were compared between cryoprecipitate and HFC groups using the unpaired t-test for normally distributed outcomes with approximately equal variation within the two groups. The Welch test was performed for normally distributed variables without equal spread of data values. Non-normally distributed outcomes were compared between groups using the Mann-Whitney test. For values below the limit of detection, the lower limit of the detection range was used as a substitute. P-values <0.05 were considered statistically significant.

## Results

### Coagulation factor concentrations and thrombin generation potential of individual cryoprecipitate bags and HFC vials

#### Procoagulant activation markers/components/activity

In addition to fibrinogen, cryoprecipitate was found to contain VWF, FVIII, FXIII, fibronectin, alpha-2 antiplasmin, TAT, PMPs, prothrombin fragment 1+2, and FPA; HFC contained only fibrinogen, FXIII, and trace amounts of VWF, fibronectin and FPA. Levels of each of these components in cryoprecipitate and HFC are shown in S1 Table. Mean concentrations of fibrinogen (Clauss) and FXIII in cryoprecipitate were significantly lower than in HFC. The mean level of FPA was also lower in cryoprecipitate compared with HFC, although this was not statistically significant. The molar ratio of FPA to fibrinogen was, however, significantly higher for cryoprecipitate than for HFC. Likewise, concentrations of VWF, FVIII, fibronectin, alpha-2 antiplasmin, TAT, prothrombin fragment 1+2, and PMP activity were also significantly higher in cryoprecipitate *vs*. HFC. Moreover, levels of FVIII, alpha-2 antiplasmin, TAT, prothrombin fragment 1+2, and PMP activity were below the limit of detection in HFC.

**Fig 1:**
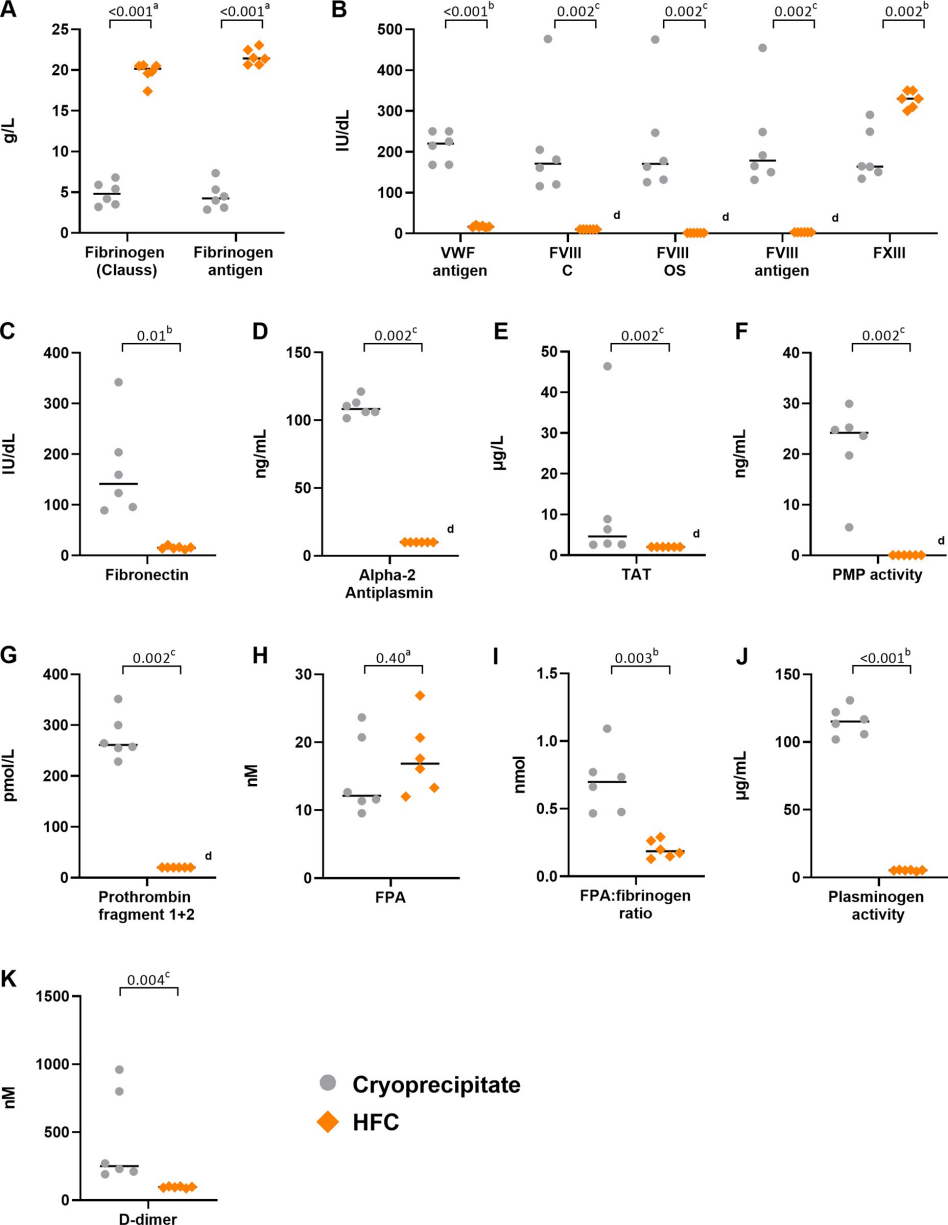
Cryoprecipitate contains more variable coagulation factor and protein concentrations than HFC. Individual data points of coagulation factor assays for cryoprecipitate (n = 6) and HFC (n = 6) for fibrinogen (Clauss) and fibrinogen antigen (**A**); VWF antigen, FVIII, FVIII OS, FVIII antigen, and FXIII (**B**); fibronectin (**C**); alpha-2 antiplasmin (**D**); TAT (**E**); PMP activity (**F**); prothrombin fragment 1+2 (**G**); FPA (**H**); molar ratio of FPA to fibrinogen (**I**); plasminogen activity (**J**); and D-dimer (**K**). ^a^Analysis using unpaired t-test. ^b^Analysis using Welch test. ^c^Analysis using Mann-Whitney test. ^d^Levels below the limit of detection; the lower limit of the detection range was used as a substitute value. C, chromogenic; FPA, fibrinopeptide A; FVIII, factor VIII; FXIII, factor XIII; HFC, human fibrinogen concentrate; OS, one-stage; PMP, platelet-derived microparticle; TAT, thrombin anti-thrombin; VWF, von Willebrand factor.

Individual data points for procoagulant factor concentrations in S1 Table are shown visually in Fig 1A–1I; these demonstrate that cryoprecipitate bags contain highly variable levels of procoagulant factors compared with HFC vials. In particular, levels were particularly variable for FVIII and FXIII (Fig 1B), fibronectin (Fig 1C), and TAT (Fig 1E) in cryoprecipitate. PMP activity and prothrombin fragment 1+2 levels were also highly variable in cryoprecipitate (Fig 1F and 1G, respectively). In contrast, HFC contained more consistent procoagulant levels (Fig 1A–1H), with the exception of FPA, which had comparable variability to that in cryoprecipitate (Fig 1H). However, the molar ratio of FPA to fibrinogen was more consistent for HFC than for cryoprecipitate (Fig 1I).

#### Plasminogen and D-dimer fragments

Cryoprecipitate contained a significantly higher level of plasminogen activity compared with HFC, as well as significantly higher levels of D-dimer (S1 Table). Individual concentrations of these factors/fragments were highly variable in cryoprecipitate (Fig 1J and 1K).

### Coagulation factor content and thrombin generation potential per standard dose of cryoprecipitate and HFC

#### Procoagulant activation markers/components/activity

**Fig 2:**
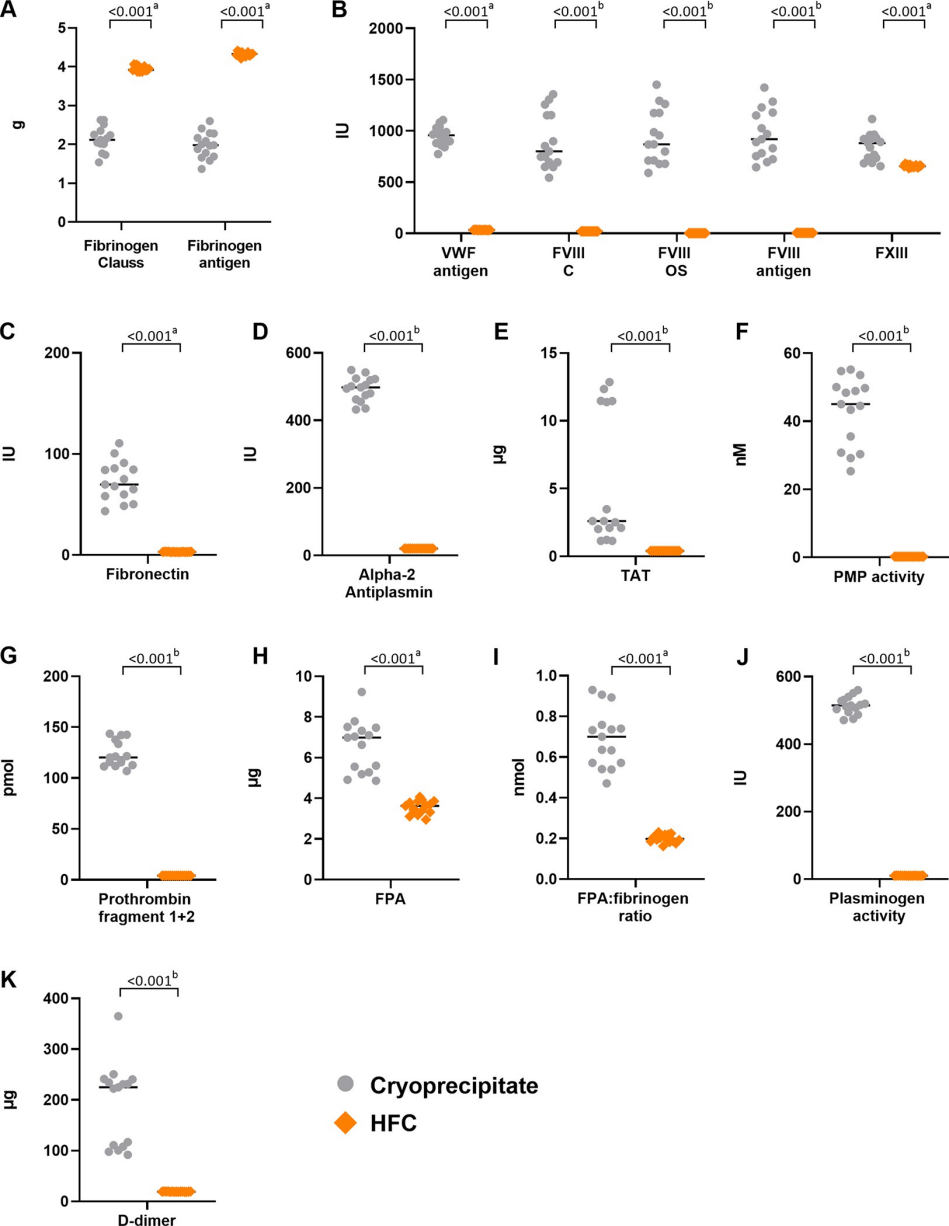
A standard dose of cryoprecipitate contains more variable levels of coagulation factors and proteins than a standard dose of HFC. Individual data points of coagulation factor assays for cryoprecipitate (n = 6) and HFC (n = 6) for fibrinogen (Clauss) and fibrinogen antigen (**A**); VWF antigen, FVIII, FVIII OS, FVIII antigen, and FXIII (**B**); fibronectin (**C**); alpha-2 antiplasmin (**D**); TAT (**E**); PMP activity (**F**); prothrombin fragment 1+2 (**G**); FPA (**H**); molar ratio of FPA to fibrinogen (**I**); plasminogen activity (**J**); and D-dimer (**K**). ^a^Analysis using Welch test. ^b^Analysis using Mann-Whitney test. ^c^Levels per cryoprecipitate bag/HFC vial were below the limit of detection; the lower limit of the detection range was used as a substitute value for the standard dose calculation. C, chromogenic; FPA, fibrinopeptide A; FVIII, factor VIII; FXIII, factor XIII; HFC, human fibrinogen concentrate; OS, one-stage; PMP, platelet-derived microparticle; TAT, thrombin anti-thrombin; VWF, von Willebrand factor.

The results per standard dose were largely similar to those shown for individual cryoprecipitate bags and HFC batches. Per standard cryoprecipitate dose (10 U), the mean fibrinogen (Clauss) level was approximately half that in a standard 4 g dose of HFC (2.14 *vs*. 3.95 g, respectively; p<0.001), whereas the mean FXIII level was higher in a standard cryoprecipitate dose compared with HFC (p<0.001; S2 Table). Mean cryoprecipitate levels per standard dose were also significantly higher for levels of VWF, FVIII, fibronectin, alpha-2 antiplasmin, TAT, prothrombin fragment 1+2, FPA, the molar ratio of FPA to fibrinogen, and PMP activity, compared with HFC (S2 Table). Individual datapoints for the 15 permutations of two cryoprecipitate bags and four HFC vials (to simulate standard dosing [10 U *vs*. 4 g, respectively]) show that cryoprecipitate doses contain highly variable levels of procoagulants compared with HFC, which had more consistent dosing levels (Fig 2A–2I).

#### Plasminogen and D-dimer fragments

The mean level of plasminogen activity was significantly higher in a standard dose of cryoprecipitate compared with a standard dose of HFC (S2 Table and Fig 2J). Levels of D-dimer were also significantly higher and more variable in a standard dose of cryoprecipitate compared with HFC (Fig 2K).

## Discussion

This study provides a comprehensive characterization of the largely undocumented coagulation-relevant constituents of cryoprecipitate and compares the composition variability to that of a highly purified and pathogen-inactivated HFC. In this comparison, cryoprecipitate contained lower concentrations of fibrinogen and FXIII compared with HFC, while levels of these coagulation factors were highly variable between cryoprecipitate bags, translating to variable dosing in a standard dose of cryoprecipitate for replacement therapy. In comparison, HFC coagulation factor content was consistent between vials, translating to reproducible dosing predictability. Cryoprecipitate also contained variable and often high concentrations of the acute phase proteins VWF and FVIII, which may provide variable hemostatic efficacy and increase the potential risk of TEs.

The four-fold higher fibrinogen content in HFC compared with cryoprecipitate in the current study (19.73 *vs*. 4.83 g/L, respectively; p<0.001) supports the results of a separate comparison of another fibrinogen concentrate (*RiaSTAP*) *vs*. cryoprecipitate (21.6 *vs*. 6.8 g/L, respectively) [17]. Despite the lower fibrinogen content in cryoprecipitate compared with *RiaSTAP*, it was suggested that cryoprecipitate may be a superior source of fibrinogen due to the reduced susceptibility of cryoprecipitate clots to fibrinolysis and the formation of a more homogenous fibrin network [17]. However, this interpretation may be related to the composition of *RiaSTAP*, which contains lower amounts of clot-stabilizing FXIII compared with the tested HFC (165 *vs*. 585 U per 3 g dose) [18], which may have affected fibrinolytic stability. Therefore, the suggestion that cryoprecipitate may be a superior source of fibrinogen compared with HFC, may not be applicable to all fibrinogen concentrates. Additionally, we demonstrate that a standard dose of HFC contains approximately double the amount of fibrinogen compared with a standard cryoprecipitate dose (3.95 *vs*. 2.14 g, respectively; p<0.001). Also, in a standard dose of cryoprecipitate, fibrinogen levels varied by >1 g, whereas fibrinogen levels per standard dose of HFC differed by only 0.2 g, reflecting the more accurate and consistent fibrinogen dosing potential of HFC. As a source of fibrinogen, the tested HFC may, therefore, be superior to cryoprecipitate.

Another advantage of the tested HFC over cryoprecipitate appears to be significantly higher and more consistent FXIII levels. Deficiency of fibrin-stabilizing FXIII can result in hyperfibrinolysis and severe episodes of bleeding, which can be successfully treated with direct FXIII replacement using cryoprecipitate, plasma-derived FXIII, recombinant FXIII, or FXIII-containing HFC [19]. Mean FXIII levels in the tested HFC have also been reported as higher than those for other fibrinogen concentrates, i.e., *RiaSTAP* and *FibClot* (3.9, 1.1, and 2.1 U/mL, respectively) [18]. However, when we estimated FXIII concentrations per standard dose, mean FXIII levels were significantly higher in a standard dose of cryoprecipitate, again with cryoprecipitate showing more variability. These results reflect those from the FORMA-05 RCT, where FXIII levels were comparable between HFC and cryoprecipitate groups experiencing major bleeding whilst undergoing CRS for pseudomyxoma peritonei [13]. As FXIII is not an acute phase protein that requires prompt replenishment during surgical bleeding, satisfactory supplementation with FXIII can be obtained using the tested HFC [13].

Unlike FXIII, FVIII and VWF are acute phase reactants, and their activities were also comparable between HFC and cryoprecipitate treatment groups in FORMA-05 [13]. As acute phase reactants, FVIII and VWF levels can increase during inflammation and endothelial disruption. As such, additional FVIII replenishment is not established in patients undergoing surgery or experiencing traumatic bleeding, due to elevated thromboembolic risk [20,21]. Likewise, VWF replacement in acute bleeding other than in VWF deficiencies is not clinically established due to association with a high risk of venous thrombosis [22]. Reflecting this, both FIBRES and FORMA-05 reported higher TE rates in patients who received cryoprecipitate (9.6% and 30.4%, respectively) compared with those who received HFC (7.0% and 0.0%, respectively) [12,13]. Due to this apparent lower risk of TEs, HFC may be preferable to cryoprecipitate in patients at increased risk of TEs.

The consistently low levels of fibronectin shown in HFC compared with cryoprecipitate in the current study are also advantageous in terms of lowering the thromboembolic risk. Although fibronectin is vital to control bleeding in patients with fibrinogen deficiencies [23], high fibronectin levels have been associated with venous thromboembolism [24]. The lower and less variable fibronectin levels seen in HFC may, therefore, be more clinically advantageous for treatment of acute bleeding.

Platelet activation to control acute bleeding leads to the formation of PMPs; membrane-bound vesicles containing procoagulant proteins and microRNAs that modulate gene expression through post-transcriptional mechanisms [25–27]. Like other cryoprecipitate procoagulant constituents, PMP activity in the current study was higher and more variable compared with HFC. PMPs have been associated with high procoagulant activity, but also play roles in inflammation, angiogenesis, and immunity [26]. Their physiological role in thrombosis and hemostasis, as well as their clinical relevance, remains controversial [28,29].

Cryoprecipitate contains wide coagulation factor variability due to the natural variability of coagulation factor levels between donors [2,6,30]. Therefore, use of cryoprecipitate may lead to inaccurate dosing for the treatment of acquired fibrinogen deficiencies, especially when utilizing thromboelastometry techniques for point-of-care determination and adapted replacement strategies as recommended by recent guidelines [31–34]. Another disadvantage of cryoprecipitate is that it contains inhibitors of coagulation and FDPs. Plasminogen, a key effector of fibrinolysis [35] is found in cryoprecipitate and not in HFC. Another protein found at high levels in cryoprecipitate and not HFC is alpha-2 antiplasmin. Due to the variability of plasminogen activity and alpha-2 antiplasmin levels in cryoprecipitate, their combined effect on hemostasis is uncertain; ultimately, this translates into variable hemostatic efficacy. Cryoprecipitate also had high and variable D-dimer and FPA levels, and a molar ratio of FPA to fibrinogen. High D-dimer levels are suggestive of fibrinolytic system activation, which may have knock-on effects in cryoprecipitate/fibrinogen concentrate recipients; FPA has a very short half-life (3–5 minutes) and so may have little impact on blood clot stability [18]. The higher and more variable molar ratio of FPA to fibrinogen in cryoprecipitate suggest that a greater proportion of fibrinogen in cryoprecipitate is already activated and may be present as fibrin monomers. It would, therefore, be reasonable to imply that cryoprecipitate may contain significantly more spontaneously polymerizable fibrin(ogen) than HFC, which would not be under the control of *in vivo* thrombin and so would impair coagulation.

Prothrombin fragment 1+2 and TAT were also significantly higher and more variable in cryoprecipitate compared with HFC. Related to elevated FVIII content, which has an influence on thrombin generation, increasing concentrations of exogenous fibrinogen from cryoprecipitate has been shown to shorten clotting times in fibrinogen-deficient plasma, whereas the addition of *RiaSTAP* can result in progressive lengthening of clotting time [17]. However, these spiking experiments were performed in fibrinogen-deficient plasma and, therefore, were not reflective of the ongoing acute phase dynamics present during acute bleeding in a clinical setting. In a more relevant clinical study of patients experiencing acute bleeding whilst undergoing CRS for pseudomyxoma peritonei, clot firmness increased after administration of both HFC and cryoprecipitate, but to a significantly greater extent with HFC, as assessed by FIBTEM A20 (measured by rotational thromboelastometry [p = 0.003]) [13]. HFC also demonstrated noninferior efficacy to cryoprecipitate (p = 0.0095), with a significantly greater mean increase in plasma fibrinogen level (0.78 *vs*. 0.35 g/L; p<0.0001), despite not containing any VWF, FVIII, alpha-2 antiplasmin, TAT, PMPs, or prothrombin fragment 1+2. Furthermore, HFC was available in the operating room 46 minutes faster than cryoprecipitate, meaning that treatment was initiated sooner than was possible with cryoprecipitate [13].

The tested HFC has demonstrated efficacy and a favorable safety profile in other studies [36–38]. In Phase 3 clinical trials, overall hemostatic efficacy in the treatment of bleeding episodes in patients with congenital fibrinogen deficiency was rated as 98.9% and 100% successful in adult/adolescent and pediatric patients, respectively, and was 100% successful for surgical prophylaxis in all age groups [37,38]. The consistent HFC fibrinogen content, and therefore, the ability to tailor treatment by calculating specific amounts of fibrinogen required to attain pre-determined target levels (100 and 150 mg/dL for minor and major bleeding/surgery, respectively), likely contributed to the observed hemostatic success of HFC.

Limitations of our study include that only a single source of cryoprecipitate was used and that no measurements of additional functional testing were performed. Also, plasminogen was the only anticoagulant measured as Protein C, Protein S, and antithrombin III are not enriched in cryoprecipitate [39]; therefore, a significant influence on their coagulation properties is not expected. Furthermore, assays were not standardized between cryoprecipitate and HFC as we aimed only to show *in vitro* variability associated with cryoprecipitate compared with HFC. Direct comparison of differences between cryoprecipitate and HFC should, therefore, be interpreted with caution. Further in-depth studies are needed to explore the differences reported herein and determine their impact on the kinetics of coagulation.

The clinical relevance of our findings has not yet been investigated, though other studies discussed above suggest that HFC has noninferior efficacy and improved safety compared with cryoprecipitate. It is hoped that future studies, like the ongoing Phase 3 trial investigating the efficacy and safety of intraoperative HFC *vs*. cryoprecipitate in individuals undergoing major spinal or abdominal surgery [40], will shed more light on clinical outcomes with different fibrinogen sources. Future studies would be beneficial if they reported coagulation factor levels post-surgery (in addition to intraoperative levels), as with FORMA-05, to better understand the efficacy of different fibrinogen sources. However, assessing safety parameters in clinical trials will be difficult; the contribution of individual parameters to thromboembolic risk, or the clinical relevance of each parameter remain to be determined; such studies will be difficult to execute.

Our findings demonstrate that, compared with cryoprecipitate, the tested HFC is a more reliable and consistent source for accurate dosing of fibrinogen and FXIII due to significantly higher and more standardized concentrations. The advantages of HFC over cryoprecipitate translate to accurate dosing, faster preparation/administration time, evidence of clinical efficacy, and improved safety. Furthermore, HFC can be stored in clinical areas at room temperature and does not require frozen storage, unlike cryoprecipitate. In addition to fibrinogen and FXIII, we show that cryoprecipitate also contains VWF, FVIII, fibronectin, alpha-2 antiplasmin, TAT, PMPs, prothrombin fragment 1+2, FPA, plasminogen, and D-dimer at variable levels. The clinical effect of variable pro- and anticoagulant levels remain unclear, but may interfere with the therapeutic aim of treating acquired fibrinogen deficiency. In contrast, HFC contains far lower and less variable levels of these factors. A novel finding was that cryoprecipitate contains substantial and variable PMP and plasminogen activity.

## Conclusion

Cryoprecipitate treatment does not appear to be a reliable choice for goal-directed therapy in bleeding management. Cryoprecipitate may also potentially elevate VWF and FVIII to prothrombotic levels. The varying levels of prothrombotic and anticoagulative elements in cryoprecipitate may impair treatment goals and have variable risks toward thrombosis or the continuation of bleeding in critical patients; high PMP activity may strongly contribute to this. In comparison, treatment with HFC enables a tailored and accurate fibrinogen replacement strategy, especially in the setting of managed care and responsible patient blood management concepts in both trauma and surgery.

## Supporting information

S1 File Supplementary materials and methods. (DOCX)

S1 Table Coagulation factor and protein concentrations in cryoprecipitate and HFC. (DOCX)

S2 Table Estimated coagulation factor and protein content per standard dose of cryoprecipitate and HFC. (DOCX)
