# Supplementary material for: Differences in coagulation-relevant parameters: Comparing cryoprecipitate and a human fibrinogen concentrate
Source: PLoS One. 2023 Aug 30;18(8):e0290571. doi: 10.1371/journal.pone.0290571 (PMC10468048; doi:10.1371/journal.pone.0290571)
Supplement: S1 File — (DOCX) [file pone.0290571.s001.docx]

**Differences in coagulation-relevant parameters: Comparing cryoprecipitate and a human fibrinogen concentrate**

Sophia Stanford^1^, Ashok Roy^1^*, Tom Cecil^1^, Oliver Hegener^2^, Petra Schulz^3^, Anna Turaj^4^, Sean Lim^4^, and Emily Arbuthnot^1^

^1^ Peritoneal Malignancy Institute, Basingstoke and North Hampshire Hospital, Basingstoke, UK

^2^ Octapharma AG, Lachen, Switzerland

^3^ Octapharma GmbH, Vienna, Austria

^4^ Centre for Cancer Immunology, Faculty of Medicine, University of Southampton, MP127 University Hospital Southampton, Southampton, UK

***Corresponding author:**

[ashok.roy@hhft.nhs.uk](mailto:ashok.roy@hhft.nhs.uk) (AR)

# **Supporting information**

## **Supplementary materials and methods**

### **Assay methods**

### **Procoagulant activation markers/components/activity**

Clauss fibrinogen activity was measured as previously described [1, 2]. Fibrinogen antigen levels were measured using the LIAPHEN Fibrinogen Assay (Hyphen BioMed; Neuville-sur-Oise, France) and by nephelometry (Siemens Healthcare; Erlangen, Germany) for cryoprecipitate and human fibrinogen concentrate (HFC; *Fibryga*, Octapharma), respectively. Von Willebrand factor (VWF) antigen levels were measured using the HemosIL AcuStar VWF:Ag Kit (Werfen; Barcelona, Spain) and the VWF-Enzym-Immunoassay-Testkit (Asserachrom VWF:Ag; Diagnostica Stago; Asnières-sur-Seine, France), for cryoprecipitate and HFC, respectively. Factor VIII (FVIII) levels were determined using the Coamatic Factor VIII (Quadratech Diagnostics; Lewes, UK) automated chromogenic assay with FVIII-deficient plasma (Siemens Healthcare; Erlangen, Germany). The FVIII one-stage (OS) automated clotting-based assay, measured using activated partial thromboplastin time, was also used to determine FVIII levels. FVIII OS results were interpreted from a reference curve obtained from pre-diluted standards of human plasma mixed with FVIII-deficient plasma. The time taken to clot was inversely proportional to FVIII concentration. FVIII antigen levels were measured using the Enzym-Immunoassay-VIII:Ag-Testkit (Asserachrom VIII:Ag; Diagnostica Stago; Asnières-sur-Seine, France). Factor XIII (FXIII) levels were determined using the Berichrom FXIII Chromogenic Ammonia Release Assay (Siemens Healthcare; Erlangen, Germany), as previously described[3], and the TECHNOZYM Fibronectin Test (Technoclone; Wien, Austria) was used to determine the amount of intact, uncleaved fibronectin. Alpha-2 antiplasmin levels were measured using the Berichrom α2-Antiplasmin Assay Kit (Siemens Healthcare; Erlangen, Germany).

Platelet-derived microparticle (PMP) activity was measured using the ZYMUPHEN MP-ACTIVITY Kit (Hyphen Biomed; Neuville-sur-Oise, France), according to the manufacturer’s microtiter plate format, and thrombin anti-thrombin complex (TAT) levels were measured using the Enzygnost TAT Micro ELISA Kit (Siemens Healthcare; Erlangen, Germany). A sandwich enzyme-linked immunosorbent assay (ELISA) for prothrombin fragment 1+2 was performed using the Enzygnost F1+2 (monoclonal) ELISA Kit (Siemens Healthcare; Erlangen, Germany) and fibrinogen-free-fibrinopeptide A (FPA) was measured in a competitive ELISA (microtiter plate format) on bentonite adsorbed human plasma with the ZYMUTEST FPA Kit (Hyphen Biomed; Neuville-sur-Oise, France).

# **References**

1. Clauss A. [Rapid physiological coagulation method in determination of fibrinogen]. Acta Haematol*.* 1957;17(4):237-46. doi: 10.1159/000205234

2. Schulz PM, Gehringer W, Nöhring S, Müller S, Schmidt T, Kekeiss-Schertler S, et al. Biochemical characterization, stability, and pathogen safety of a new fibrinogen concentrate (fibryga). Biologicals*.* 2018;52:72-77. doi: 10.1016/j.biologicals.2017.12.003

3. Lawrie AS, Green L, Mackie IJ, Liesner R, Machin SJ, and Peyvandi F. Factor XIII – an under diagnosed deficiency – are we using the right assays? J Thromb Haemost*.* 2010;8(11):2478-2482. doi: 10.1111/j.1538-7836.2010.04028.x
