# Supplementary material for: Differences in coagulation-relevant parameters: Comparing cryoprecipitate and a human fibrinogen concentrate
Source: PLoS One. 2023 Aug 30;18(8):e0290571. doi: 10.1371/journal.pone.0290571 (PMC10468048; doi:10.1371/journal.pone.0290571)
Supplement: S1 Table — (DOCX) [file pone.0290571.s002.docx]

**S1 Table. Coagulation factor and protein concentrations in cryoprecipitate and HFC.**

| **Assay** | **Cryoprecipitate (n=6)** | | **HFC (n=6)** | | **P** |
| --- | --- | --- | --- | --- | --- |
|  | **Mean ± SD** | **Median (Range)** | **Mean ± SD** | **Median (Range)** |  |
| **Procoagulant activation markers/components/activity** |  |  |  |  |  |
| Fibrinogen (Clauss) [g/L]^a^ | 4.83 ± 1.43 | 4.80 (3.20‒6.80) | 19.73 ± 1.22 | 20.15 (17.40‒20.60) | <0.001 |
| Fibrinogen antigen [g/L]^a^ | 4.52 ± 1.65 | 4.24 (2.86‒7.35) | 21.62 ± 0.97 | 21.44 (20.65‒23.06) | <0.001 |
| VWF antigen [IU/dL]^b^ | 212.75 ± 37.12 | 220.00 (168.00‒250.00) | 17.00 ± 2.61 | 16.50 (14.00‒21.00) | <0.001 |
| FVIII C [IU/dL]^c^ | 210.03 ± 135.01 | 171.00 (116.10‒476.50) | <10.00 ± n/a^d^ | <10.00 (n/a) | 0.002 |
| FVIII OS [IU/dL]^c^ | 220.03 ± 132.25 | 170.60 (125.50‒475.00) | <1.50 ± n/a^d^ | <1.50 (n/a) | 0.002 |
| FVIII antigen [IU/dL]^c^ | 223.67 ± 120.35 | 178.50 (131.50‒454.90) | <3.30 ± n/a^d^ | <3.30 (n/a) | 0.002 |
| FXIII [IU/dL]^b^ | 192.17 ± 62.70 | 164.05 (134.20‒290.70) | 328.33 ± 20.41 | 330.00 (300.00‒350.00) | 0.002 |
| Fibronectin [µg/mL]^b^ | 168.97 ± 94.99 | 141.56 (89.10‒342.21) | 15.58 ± 3.11 | 15.20 (11.70‒20.70) | 0.01 |
| Alpha-2 Antiplasmin [IU/dL]^c^ | 109.78 ± 6.84 | 108.35 (101.60‒121.20) | <10.00 ± n/a^d^ | <10.00 (n/a) | 0.002 |
| TAT [µg/L]^c^ | 11.62 ± 17.22 | 4.62 (2.58‒46.39) | <2.00 ± n/a^d^ | <2.00 (n/a) | 0.002 |
| PMP activity [nM]^c^ | 21.48 ± 8.46 | 24.20 (5.55‒29.95) | <0.05 ± n/a^d^ | <0.05 (n/a) | 0.002 |
| Prothrombin fragment 1+2 [pmol/L]^c^ | 276.17 ± 43.42 | 261.00 (228.50‒351.40) | <20.00 ± n/a^d^ | <20.00 (n/a) | 0.002 |
| FPA [ng/mL]^a^ | 14.94 ± 5.79 | 12.15 (9.57‒23.67) | 17.77 ± 5.44 | 16.85 (12.00‒26.90) | 0.40 |
| FPA:fibrinogen ratio [µmol]^b^ | 0.0007 ± 0.0002 | 0.0007 (0.0005‒0.0011) | 0.0002 ± 0.0001 | 0.0002 (0.0001‒0.0003) | 0.003 |
| **Plasminogen and D-dimer fragments** |  |  |  |  |  |
| Plasminogen activity [IU/dL]^b^ | 115.15 ± 10.60 | 115.15 (101.90‒130.80) | 5.25 ± 0.46 | 5.30 (4.40‒5.70) | <0.001 |
| D-dimer [ng/mL]^c^ | 443.33 ± 343.03 | 250.00 (190.00‒960.00) | 96.12 ± 5.77 | 97.15 (87.20‒101.70) | 0.004 |

^a^Analysis using unpaired t-test.

^b^Analysis using Welch test.

^c^Analysis using Mann-Whitney test.

^d^Levels below the limit of detection; the lower limit of the detection range was used as a substitute value.

C, chromogenic; FPA, fibrinopeptide A; FVIII, factor VIII; FXIII, factor XIII; HFC, human fibrinogen concentrate; n/a, not applicable; OS, one-stage; SD, standard deviation; PMP, platelet-derived microparticle; TAT, thrombin anti-thrombin; VWF, von Willebrand factor.
