# Supplementary material for: Differences in coagulation-relevant parameters: Comparing cryoprecipitate and a human fibrinogen concentrate
Source: PLoS One. 2023 Aug 30;18(8):e0290571. doi: 10.1371/journal.pone.0290571 (PMC10468048; doi:10.1371/journal.pone.0290571)
Supplement: S2 Table — (DOCX) [file pone.0290571.s003.docx]

**S2 Table. Estimated coagulation factor and protein content per standard dose of cryoprecipitate and HFC.**

| **Assay** | **Cryoprecipitate (n=6)** | | **HFC (n=6)** | | **P** |
| --- | --- | --- | --- | --- | --- |
|  | **Mean ± SD** | **Median (Range)** | **Mean ± SD** | **Median (Range)** |  |
| **Procoagulant activation markers/components/activity** |  |  |  |  |  |
| Fibrinogen (Clauss) [g]^a^ | 2.14 ± 0.32 | 2.12 (1.54‒2.63) | 3.95 ± 0.07 | 3.92 (3.87‒4.07) | <0.001 |
| Fibrinogen antigen [g]^a^ | 1.98 ± 0.33 | 1.98 (1.37‒2.60) | 4.32 ± 0.06 | 4.33 (4.21‒4.42) | <0.001 |
| VWF antigen [IU]^a^ | 948.69 ± 93.00 | 958.00 (773.98‒1106.50) | 34.00 ± 1.56 | 34.00 (31.00‒36.50) | <0.001 |
| FVIII C [IU]^b^ | 900.53 ± 270.68 | 800.28 (543.74‒1357.59) | 20.00 ± n/a^c^ | 20.00 (n/a) | <0.001 |
| FVIII OS [IU]^b^ | 946.99 ± 266.66 | 868.90 (591.25‒1451.33) | 3.00 ± n/a^c^ | 3.00 (n/a) | <0.001 |
| FVIII antigen [IU]^b^ | 967.12 ± 239.31 | 919.22 (646.53‒1423.29) | 6.60 ± n/a^c^ | 6.60 (n/a) | <0.001 |
| FXIII [IU]^a^ | 846.67 ± 130.80 | 880.56 (655.86‒1116.40) | 656.67 ± 12.20 | 655.00 (635.00‒680.00) | <0.001 |
| Fibronectin [mg]^a^ | 73.01 ± 19.66 | 69.74 (43.34‒110.56) | 3.12 ± 0.19 | 3.14 (2.80‒3.40) | <0.001 |
| Alpha-2 Antiplasmin [IU]^b^ | 492.95 ± 36.10 | 497.52 (432.18‒549.16) | 20.00 ± n/a^c^ | 20.00 (n/a) | <0.001 |
| TAT [µg]^b^ | 5.36 ± 4.84 | 2.60 (1.14‒12.85) | 0.40 ± n/a^c^ | 0.40 (n/a) | <0.001 |
| PMP activity [nM]^b^ | 42.97 ± 10.11 | 45.00 (25.31‒55.19) | 0.20 ± n/a^c^ | 0.20 (n/a) | <0.001 |
| Prothrombin fragment 1+2 [pmol]^b^ | 123.57 ± 12.77 | 120.23 (106.91‒143.57) | 4.00 ± n/a^c^ | 4.00 (n/a) | <0.001 |
| FPA [µg]^a^ | 6.57 ± 1.27 | 6.99 (4.86‒9.23) | 3.55 ± 0.33 | 3.63 (2.95‒4.07) | <0.001 |
| FPA:fibrinogen ratio [µmol]^a^ | 0.0007 ± 0.0001 | 0.0007 (0.0005‒0.0010) | 0.0002 ± 0.0000 | 0.0002 (0.0002‒0.0002) | <0.001 |
| **Plasminogen and D-dimer fragments** |  |  |  |  |  |
| Plasminogen activity [IU]^a^ | 514.17 ± 25.81 | 514.99 (471.26‒560.19) | 10.50 ± 0.28 | 10.45 (10.10‒10.95) | <0.001 |
| D-dimer [ng]^b^ | 191.11 ± 80.86 | 224.85 (91.71‒364.80) | 19.22 ± 0.34 | 19.16 (18.67‒19.89) | <0.001 |

^a^Analysis using Welch test.

^b^Analysis using Mann-Whitney test.

^c^Levels per cryoprecipitate bag/HFC vial were below the limit of detection; the lower limit of the detection range was used as a substitute value for the standard dose calculation.

C, chromogenic; FPA, fibrinopeptide A; FVIII, factor VIII; FXIII, factor XIII; HFC, human fibrinogen concentrate; n/a, not applicable; OS, one-stage; SD, standard deviation; PMP, platelet-derived microparticle; TAT, thrombin anti-thrombin; VWF, von Willebrand factor.
